# Association between the Framingham steatosis index and osteoarthritis: evidence of mediation by phenotypic age

**DOI:** 10.1038/s41598-026-45922-2

**Published:** 2026-04-01

**Authors:** Tianhao Guo, Weiqi Zhang, Chen Zhao, Jiangbo Bai, Yadong Yu

**Affiliations:** 1 Department of Orthopedics, Hebei Medical University Third Hospital, Shijiazhuang, 050051 Hebei China; 2 Department of Spinal Orthopedics, Hebei Medical University Third Hospital, Shijiazhuang, 050051 Hebei China; 3 Department of Prosthodontics, Peking University School and Hospital of Stomatology, Beijing, 100081 China

**Keywords:** Fatty liver, Osteoarthritis, Adult, Nutrition surveys, NHANES, Diseases, Health care, Medical research, Risk factors

## Abstract

The Framingham Steatosis Index (FSI) is a validated surrogate marker of hepatic steatosis and metabolic dysfunction. Emerging evidence suggests that metabolic abnormalities and accelerated biological aging may contribute to osteoarthritis (OA). However, whether FSI is associated with OA and whether this relationship is mediated by Phenotypic Age (PhenoAge) remain unclear. This study conducted a cross-sectional analysis of 9279 U.S. adults from the National Health and Nutrition Examination Survey, including 948 participants with OA. Weighted logistic regression models were applied to evaluate the association between FSI and OA. Restricted cubic splines and two-piecewise linear regression were used to assess nonlinear relationships and threshold effects. Subgroup analyses were performed to examine effect modification. Exploratory mediation analysis was conducted to quantify the mediating role of PhenoAge in the FSI–OA relationship. Higher FSI was significantly associated with OA (Model 3: OR = 1.18, 95% CI 1.12–1.25, *p* < 0.001), with a clear dose–response pattern across quartiles (*p* for trend < 0.001). A nonlinear association was observed with an inflection at FSI = – 1.798; OA prevalence increased steeply below this threshold. Subgroup analyses showed consistent positive associations across strata. Exploratory mediation analysis suggested that PhenoAge may partially account for this association between FSI and OA, accounting for 23.03% of the total effect. FSI was positively associated with OA prevalence in U.S. adults, and Phenotypic Age may partially account for this association. Future longitudinal studies are needed to clarify causality and determine whether improving metabolic health or slowing biological aging can reduce OA risk.

## Introduction

Osteoarthritis (OA) is the most common joint disease worldwide, characterized by progressive degeneration of articular cartilage and subchondral bone remodeling^1,2^. Traditionally attributed to “wear and tear,” OA pathogenesis is now recognized to involve metabolic and inflammatory factors^3^. Metabolic syndrome has been causally linked to OA prevalence^4,5^. Obesity and dyslipidemia contribute to a low-grade systemic inflammatory milieu, with adipose-derived cytokines and oxidative stress promoting cartilage degradation^6,7^. Multiple studies have confirmed a positive correlation between obesity and the incidence of OA^8–10^. Thus, current concepts of a “metabolic OA” phenotype emphasize systemic metabolic dysregulation as a key driver of joint degeneration, beyond mechanical loading alone.

Nonalcoholic fatty liver disease (NAFLD), recently termed metabolic dysfunction-associated steatotic liver disease (MASLD), is an ectopic fat storage condition strongly associated with obesity, insulin resistance, and metabolic syndrome^11,12^. NAFLD has a high prevalence among individuals with metabolic risk factors, and is considered a hepatic manifestation of metabolic syndrome^13^. Emerging evidence suggests that NAFLD may be linked to arthritis^14^. This implies that fatty liver burden might contribute to joint pathology, potentially via systemic inflammation or metabolic insults. Given the growing global burden of both NAFLD and OA, understanding links between hepatic steatosis and OA could inform screening and prevention strategies. The FSI is a validated index derived by Long et al., which combines demographic and clinical variables to predict hepatic steatosis^15^. This composite score has demonstrated good discrimination for NAFLD. More recently, FSI has been associated not only with liver outcomes but with extra-hepatic complications: for example, higher FSI predicted advanced cardio-renal-metabolic (CKM) syndrome in U.S. adults, and has correlated with albuminuria and kidney disease in cross-sectional studies^16,17^. These findings underscore that FSI reflects systemic metabolic dysregulation, which may also be relevant to musculoskeletal health.

Phenotype Age (PhenoAge) is an emerging indicator for measuring biological aging, developed by Levine et al^18^. Higher PhenoAge has been linked to numerous adverse health outcomes, including cardiovascular disease, diabetes, and frailty, suggesting it captures systemic physiological deterioration^19,20^. Notably, recent work has found that PhenoAge or related aging metrics can mediate the impact of metabolic factors on disease risks: for instance, part of the effect of central obesity on OA risk was shown to be transmitted through accelerated biological aging^21^.

Based on this background, we hypothesized that FSI would be positively associated with OA prevalence in U.S. adults, and that PhenoAge would partly mediate this association.

## Methods

### Data source and study population

**Fig. 1:**
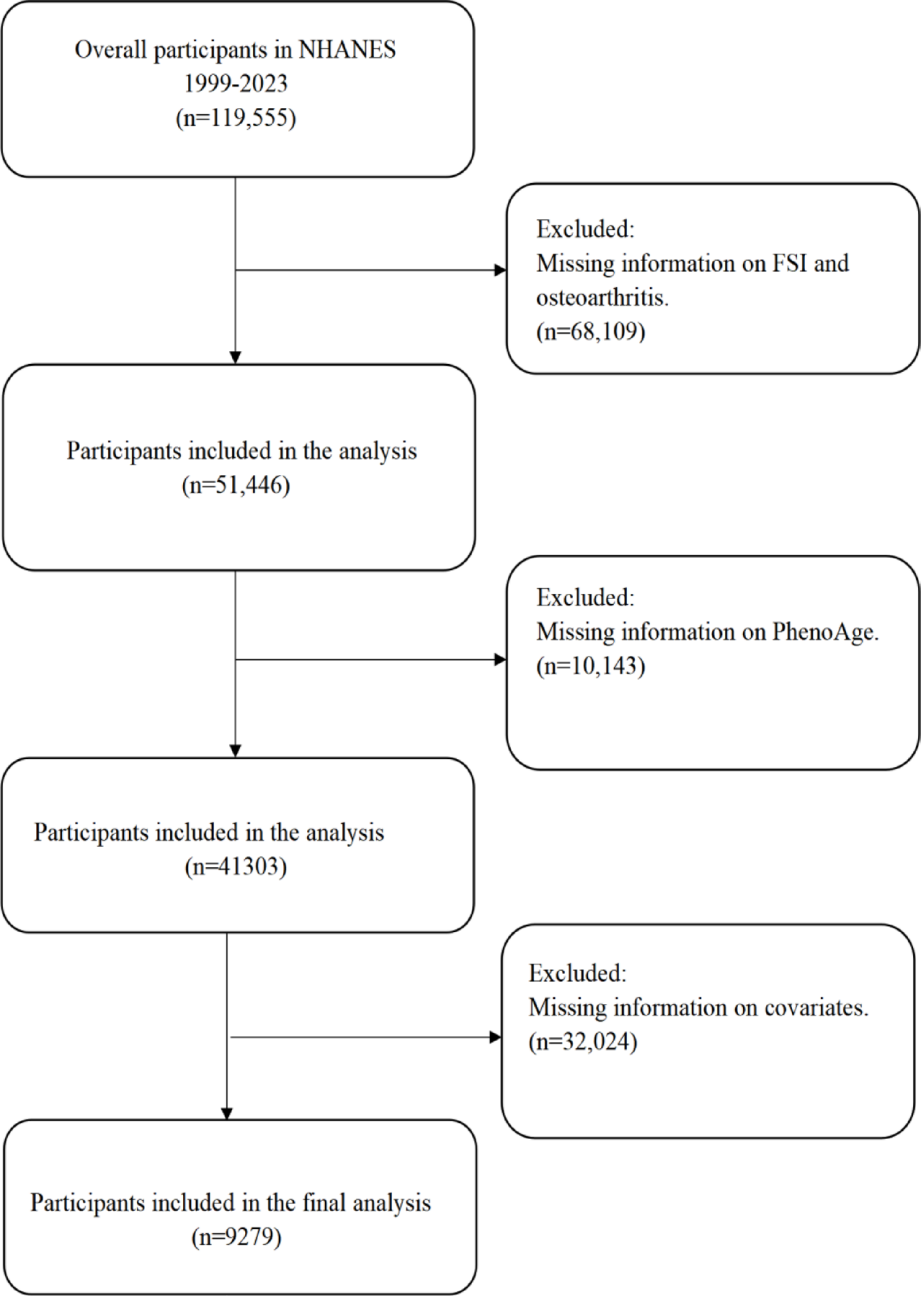
Flowchart of participant inclusion and exclusion criteria. Abbreviations: PhenoAge, Phenotypic Age; FSI, Framingham steatosis index.

We conducted a cross-sectional analysis using data from the National Health and Nutrition Examination Survey (NHANES), a program of studies designed to assess the health and nutritional status of the noninstitutionalized U.S. population. The NHANES employs a complex, multistage probability sampling design and collects detailed questionnaires, physical examinations, and laboratory measurements in each 2 years cycle. For the present analysis, we pooled multiple NHANES cycles (1999–2023) to achieve an adequate sample size. All participants provided informed consent and the survey protocol was approved by the National Center for Health Statistics institutional review board. This study followed the STROBE (Strengthening the Reporting of Observational Studies in Epidemiology) guidelines for reporting cross-sectional observational studies. Individuals lacking data for key variables were excluded from the analysis. These variables included elements required to calculate FSI, PhenoAge, BMI (body mass index), physical activity, PIR (poverty income ratio), race, marital status, cardiovascular disease, education, alcohol consumption, smoking status, hypertension, diabetes, and stroke. Finally, a total of 9279 participants were included in the study (Fig. 1).

### Study variables

The outcome was prevalent OA, defined using self-reported data. In NHANES, participants were asked whether a physician or health professional had ever told them they had arthritis, and if so to specify the type. Subjects were classified as having OA if they responded “yes” to arthritis and indicated OA as the type. This approach follows prior NHANES studies of OA^22^. We considered the OA variable as binary (yes vs. no).

The primary exposure was the FSI, a validated surrogate index for hepatic steatosis developed by Long et al^15^. In our analysis, we used the following formula: FSI =  −  7.981 + 0.011 × age (years)—0.146 × sex (female = 1, male = 0) + 0.173 × BMI (kg/m^2^) + 0.007 × triglycerides (mg/dL) + 0.593 × hypertension (yes = 1, no = 0) + 0.789 × diabetes (yes = 1, no = 0) + 1.1 × ALT: AST ratio ≥ 1.33(yes = 1, no = 0). We treated FSI both as a continuous variable and in quartiles for analyses.

PhenoAge is calculated using chronological age and nine biomarkers (serum albumin, creatinine, glucose, C-reactive protein (CRP), lymphocyte percentage, mean corpuscular volume (MCV), red cell distribution width (RDW), alkaline phosphatase (ALP), and white blood cell count). In exploratory mediation analysis we used PhenoAge as the mediator variable.

### Covariates

Potential confounders encompassed variables from demographic, examination, questionnaire, and laboratory domains. Demographic characteristics included age (age < 45 years, 45 ≤ age < 65 years, age ≥ 65 years), sex (male or female), educational attainment (less than high school, high school or equivalent, college or above), race (Non-Hispanic White, Mexican American, Non-Hispanic Black, and Other), marital status (married or cohabiting, never married, or single), and socioeconomic status measured by the PIR, categorized as low (PIR < 1.3), medium (1.3 ≤ PIR < 3.5), or high (PIR ≥ 3.5). Examination variables comprised body mass index (BMI) (BMI < 25, 25 ≤ BMI < 30, BMI ≥ 30 kg/m^2^) and physical activity. Total weekly MET-minutes were estimated following standardized procedures^23^, and participants were grouped into low (< 500 MET-min/week) and high (≥ 500 MET-min/week) physical activity levels based on national recommendations. Questionnaire-derived variables included smoking status (yes/no, with “yes” defined as ≥ 100 cigarettes consumed over a lifetime), alcohol intake (moderate drinking, heavy drinking, and mild drinking), hypertension (yes/no, defined as self-reported diagnosis, SBP ≥ 140 mmHg, DBP ≥ 90 mmHg, or use of antihypertensive medications), diabetes (yes/no, based on self-reported diagnosis, fasting plasma glucose ≥ 126 mg/dL, 2 h OGTT glucose ≥ 200 mg/dL, hemoglobin A1c ≥ 6.5%, or use of insulin or oral hypoglycemic agents), and stroke history (self-reported). Cardiovascular disease (CVD) status was determined by responses to the question: “Has a doctor or other health professional ever informed you that you have CHF, CHD, angina pectoris, myocardial infarction, or stroke?” A “yes” to any condition qualified the participant as having CVD. Laboratory measurements included liver enzymes and lipid-related markers: aspartate aminotransferase (AST), alanine aminotransferase (ALT), gamma-glutamyl transferase (GGT), and triglycerides (TG). All laboratory assays were conducted following NHANES standardized protocols.

To minimize overadjustment, the fully adjusted regression models included only selected sociodemographic, behavioral, and comorbidity-related variables, specifically educational attainment, race, marital status, PIR, smoking status, alcohol consumption, physical activity, history of stroke, and CVD. Variables such as age, sex, BMI, metabolic conditions, and laboratory markers, which are components of or closely related to the FSI, were not included in the fully adjusted models.

### Statistical methods

Because NHANES uses a stratified, multistage probability sampling framework to obtain a nationally representative sample, our analyses accounted for its design features, including primary sampling units, strata, and sample weights. All weighted estimations were performed with the survey package in R to ensure that results reflect the distribution of the U.S. non-institutionalized population and to prevent inflation of statistical significance due to complex sampling. Following NHANES analytic recommendations, we selected sample weights based on the variables and subsamples involved, giving priority to weights appropriate for smaller subpopulations. We first described baseline characteristics of the study population overall and by OA status. Differences between OA vs. non-OA groups were tested using survey-weighted t-tests for continuous variables and chi-square tests for categorical variables. To evaluate the relationship between FSI and the prevalence of OA, we employed multivariable logistic regression models. OA (yes/no) was treated as the dependent variable, and FSI was entered as both a continuous variable and in quartiles. Three models were constructed sequentially: (1) an unadjusted model; (2) a model adjusted for education level, race, marital status, and PIR; (3) a fully adjusted model that additionally included smoking status, alcohol consumption, physical activity, stroke, and CVD. We reported odds ratios (OR) and corresponding 95% confidence intervals (CI). To characterize potential nonlinear associations, restricted cubic spline (RCS) functions with four knots placed at the 5th, 35th, 65th, and 95th percentiles of FSI were incorporated into the logistic models. Threshold effects were further explored by identifying inflection points where the FSI–OA slope changed significantly. Subgroup analyses were conducted to examine potential effect modification, and interaction p-values were reported. To investigate whether phenotypic age mediated the association between FSI and OA, exploratory mediation analysis was performed with FSI as the exposure, PhenoAge as the mediator, and OA as the outcome. All statistical analyses were carried out using R software (version 4.2.0), and a two-sided *p* value < 0.05 was considered statistically significant.

## Results

### Study population characteristics

A total of 9279 participants were included, with 948 (10.22%) having OA. As shown in Table 1, OA participants were older and more often female. A higher proportion were non-Hispanic White, while fewer were Mexican American or non-Hispanic Black. Education levels were similar, but OA participants were more often widowed/divorced/separated. OA individuals had higher BMI, were more likely to smoke, had lower physical activity, and showed different alcohol-drinking patterns (more mild drinking, less heavy drinking). PIR distribution differed slightly between groups. Clinically, OA participants had lower ALT, similar AST and GGT levels, and slightly higher TG. They also had higher PhenoAge and FSI values, and were more frequently in the highest FSI quartile. Hypertension, diabetes, stroke, and cardiovascular disease were all more prevalent among OA participants.

**Table 1:** Baseline characteristics of study population.

Characteristic	Total (n = 9279)	Osteoarthritis		*p*
		No (n = 8331)	Yes (n = 948)	
ALT, mean (SE)	25.18 (0.24)	25.42 (0.26)	23.18 (0.55)	< 0.001
AST, mean (SE)	24.38 (0.21)	24.43 (0.23)	23.97 (0.47)	0.390
GGT, mean (SE)	27.88 (0.48)	28.00 (0.53)	26.89 (0.85)	0.277
TG, mean (SE)	1.39 (0.02)	1.38 (0.02)	1.47 (0.06)	0.129
PhenoAge, mean (SE)	54.12 (0.29)	52.43 (0.29)	68.46 (0.64)	< 0.001
FSI, mean (SE)	− 1.33 (0.03)	− 1.40 (0.03)	− 0.72 (0.08)	< 0.001
FSI quartile, n(%)				< 0.001
Q1	2058 (24.99)	1972 (26.72)	86 (10.29)	
Q2	2256 (24.99)	2037 (24.94)	219 (25.46)	
Q3	2472 (25.01)	2177 (24.80)	295 (26.83)	
Q4	2493 (25.00)	2145 (23.55)	348 (37.42)	
Age, n(%)				< 0.001
Age < 45	4682 (53.51)	4569 (58.26)	113 (12.99)	
45 ≤ Age < 65	3040 (33.95)	2627 (32.26)	413 (48.36)	
Age ≥ 65	1557 (12.55)	1135 (9.48)	422 (38.65)	
Gender, n(%)				< 0.001
Male	4927 (52.36)	4549 (54.05)	378 (37.95)	
Female	4352 (47.64)	3782 (45.95)	570 (62.05)	
Education, n(%)				0.451
College or above	5826 (68.93)	5178 (68.71)	648 (70.76)	
Less than high school	1478 (9.65)	1370 (9.81)	108 (8.31)	
High school or equivalent	1975 (21.42)	1783 (21.48)	192 (20.93)	
Smoking status, n(%)				< 0.001
Yes	4430 (46.87)	3930 (45.88)	500 (55.32)	
No	4849 (53.13)	4401 (54.12)	448 (44.68)	
Alcohol consumption, n(%)				< 0.001
Moderate drinking	3103 (34.60)	2840 (35.25)	263 (29.04)	
Heavy drinking	1571 (15.46)	1502 (16.50)	69 (6.65)	
Mild drinking	4605 (49.94)	3989 (48.25)	616 (64.31)	
Physical activity, n(%)				< 0.001
High physical activity	6457 (72.34)	5856 (73.05)	601 (66.26)	
Low physical activity	2822 (27.66)	2475 (26.95)	347 (33.74)	
BMI, n(%)				< 0.001
BMI < 25	2885 (32.64)	2674 (33.67)	211 (23.95)	
25 ≤ BMI < 30	3170 (34.28)	2838 (34.31)	332 (33.96)	
BMI ≥ 30	3224 (33.08)	2819 (32.02)	405 (42.10)	
Race, n(%)				< 0.001
Non-Hispanic white	4699 (72.43)	4039 (70.93)	660 (85.20)	
Mexican American	1411 (7.50)	1353 (8.13)	58 (2.12)	
Non-Hispanic black	1665 (8.94)	1544 (9.38)	121 (5.19)	
Other	1504 (11.13)	1395 (11.56)	109 (7.50)	
Marital status, n(%)				< 0.001
Married or living with a partner	5739 (65.48)	5123 (65.12)	616 (68.49)	
Never married	1832 (18.83)	1777 (20.46)	55 (4.95)	
Single (widowed/divorced/separated)	1708 (15.69)	1431 (14.42)	277 (26.56)	
PIR, n(%)				0.005
PIR < 1.3	1999 (14.05)	1842 (14.45)	157 (10.65)	
1.3 ≤ PIR < 3.5	3454 (33.22)	3123 (33.50)	331 (30.82)	
PIR ≥ 3.5	3826 (52.74)	3366 (52.06)	460 (58.53)	
Hypertension, n(%)				< 0.001
Yes	3270 (31.08)	2717 (28.55)	553 (52.55)	
No	6009 (68.92)	5614 (71.45)	395 (47.45)	
Diabetes, n(%)				< 0.001
Yes	1328 (10.59)	1116 (9.61)	212 (18.95)	
No	7951 (89.41)	7215 (90.39)	736 (81.05)	
Stroke, n(%)				< 0.001
Yes	196 (1.54)	147 (1.09)	49 (5.42)	
No	9083 (98.46)	8184 (98.91)	899 (94.58)	
CVD, n(%)				< 0.001
Yes	691 (5.76)	534 (4.62)	157 (15.52)	
No	8588 (94.24)	7797 (95.38)	791 (84.48)	

Data were presented as weighted means ± SE for continuous variables or n (%) for categorical variables.

*CVD* Cardiovascular disease, *AST* Aspartate Aminotransferase, *GGT* Gamma-Glutamyl Transferase, *ALT* Alanine Aminotransferase, *TG* Triglycerides, *FSI* Framingham steatosis index, *BMI (kg/m*^*2*^*)* Body mass index, *PIR* Poverty income ratio; PhenoAge, Phenotypic Age;

### Association of FSI with OA

In unadjusted analyses (Model 1), each one‐unit increase in FSI was associated with 20% higher odds of OA (OR = 1.20, 95%CI 1.15–1.26, *p* < 0.001). The association remained stable across progressively adjusted models, including adjustment for sociodemographics (Model 2) and for additional lifestyle and comorbidity factors (Model 3), with consistent effect estimates (Model 2: OR = 1.21, 95% CI 1.15–1.27; Model 3: OR = 1.18, 95% CI 1.12–1.25; all *p* < 0.001). When FSI was categorized into quartiles (Q1 lowest to Q4 highest), there was a clear dose–response (all *p* for trend < 0.001). In the fully adjusted model (Model 3), compared to Q1 the odds of OA were 2.24-fold higher (95%CI 1.58–3.18) in Q2, 2.28-fold higher (1.57–3.29) in Q3, and 3.41-fold higher (2.39–4.88) in Q4 (all *p* < 0.001). (Table 2).

**Table 2:** Association of FSI with OA.

Characteristics	Model1		Model2		Model3	
	OR (95%CI)	*p*	OR (95%CI)	*p*	OR (95%CI)	*p*
FSI	1.20 (1.15 ~ 1.26)	< 0.001	1.21 (1.15 ~ 1.27)	< 0.001	1.18 (1.12 ~ 1.25)	< 0.001
Q1	1.00 (Reference)		1.00 (Reference)		1.00 (Reference)	
Q2	2.65 (1.88 ~ 3.73)	< 0.001	2.42 (1.72 ~ 3.42)	< 0.001	2.24 (1.58 ~ 3.18)	< 0.001
Q3	2.81 (1.96 ~ 4.02)	< 0.001	2.56 (1.79 ~ 3.67)	< 0.001	2.28 (1.57 ~ 3.29)	< 0.001
Q4	4.13 (2.97 ~ 5.73)	< 0.001	3.95 (2.83 ~ 5.52)	< 0.001	3.41 (2.39 ~ 4.88)	< 0.001
P for trend	< 0.001		< 0.001		< 0.001	

Model 1: no covariates were adjusted. Model 2 was adjusted for Education, Race, Marital status and PIR. Model 3 was adjusted for Education, Race, Marital status, PIR, Smoking status, Alcohol consumption, Physical activity, Stroke and CVD.

*CVD* Cardiovascular disease, *FSI* Framingham steatosis index, *BMI (kg/m2)* Body mass index, *PIR* Poverty income ratio, *OA* osteoarthritis.

### Nonlinear relationship and threshold effect

**Fig. 2:**
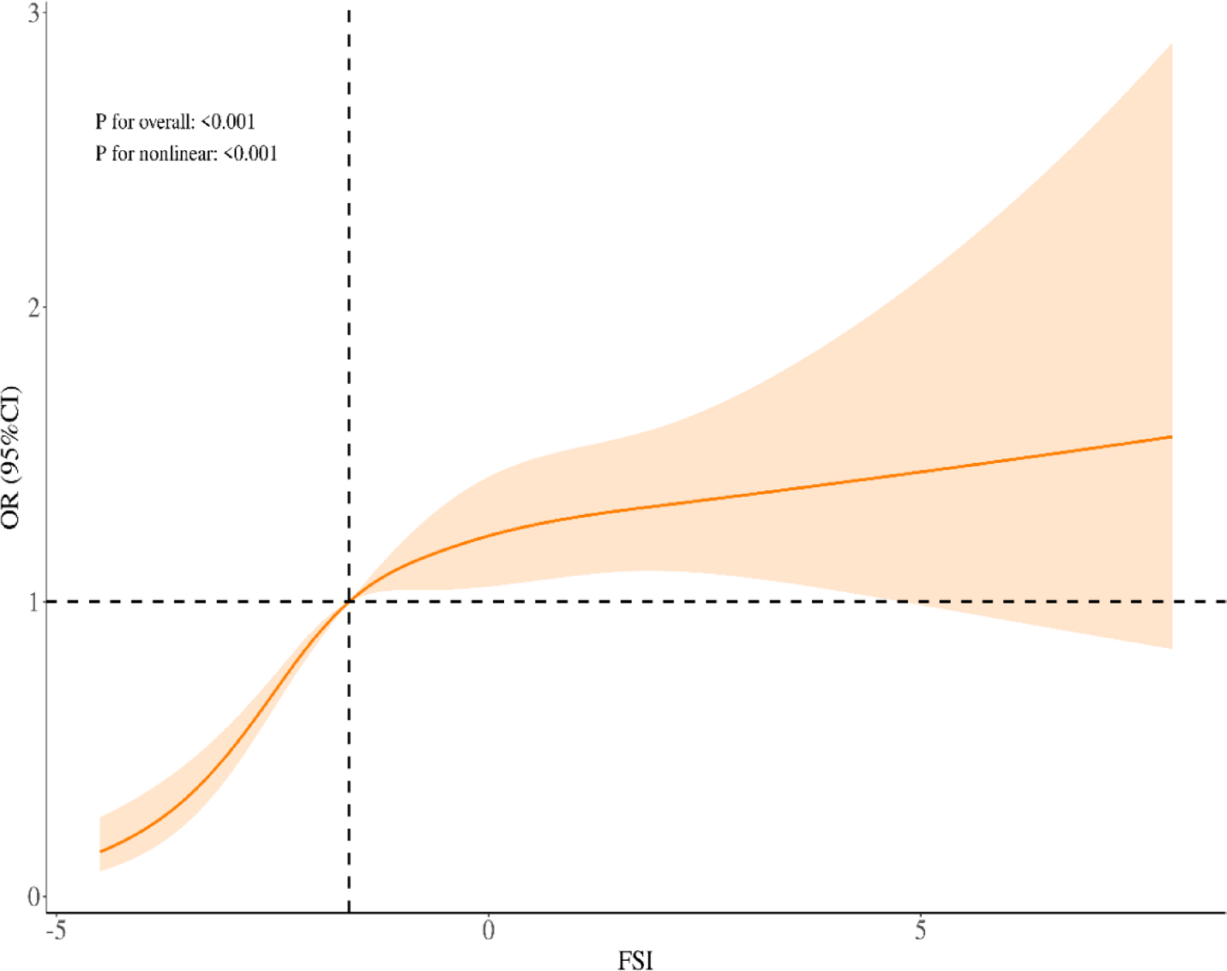
Association of FSI with OA. Solid and dashed lines represent the predicted value and 95% CI.

We further explored nonlinearity in the FSI–OA association. A two‐piecewise linear regression (Table 3) revealed an inflection at FSI = –1.798. Below this threshold, FSI was strongly associated with OA (OR = 1.97 per unit increase, 95%CI 1.54–2.52, *p* < 0.001). Above the threshold, the association flattened (OR = 1.04, 95%CI 0.98–1.10, P = 0.168). The likelihood-ratio test for nonlinearity was highly significant (*p* < 0.001). This pattern was confirmed visually in the restricted cubic spline (Fig. 2): OA prevalence rose steeply with increasing FSI, then plateaued at higher FSI values.

**Table 3:** Threshold effect analysis of the association between FSI and OA.

Outcome	OR (95% CI)	*p*
Model 1 Fitting model by standard linear regression	1.17 (1.13–1.21)	< 0.001
Model 2 Fitting model by two-piecewise linear regression		
Inflection point	− 1.798	
< − 1.798	1.97 (1.54–2.52)	< 0.001
≥ − 1.798	1.04 (0.98–1.10)	0.168
P for likelihood test		< 0.001

Education, Race, Marital status, PIR, Smoking status, Alcohol consumption, Physical activity, Stroke and CVD were adjusted.

*OR* Odds Ratio, *CI* Confidence Interval, *CVD* Cardiovascular disease, *FSI* Framingham steatosis index, *PIR* Poverty income ratio, *OA* osteoarthritis.

### Subgroup analyses

**Fig. 3:**
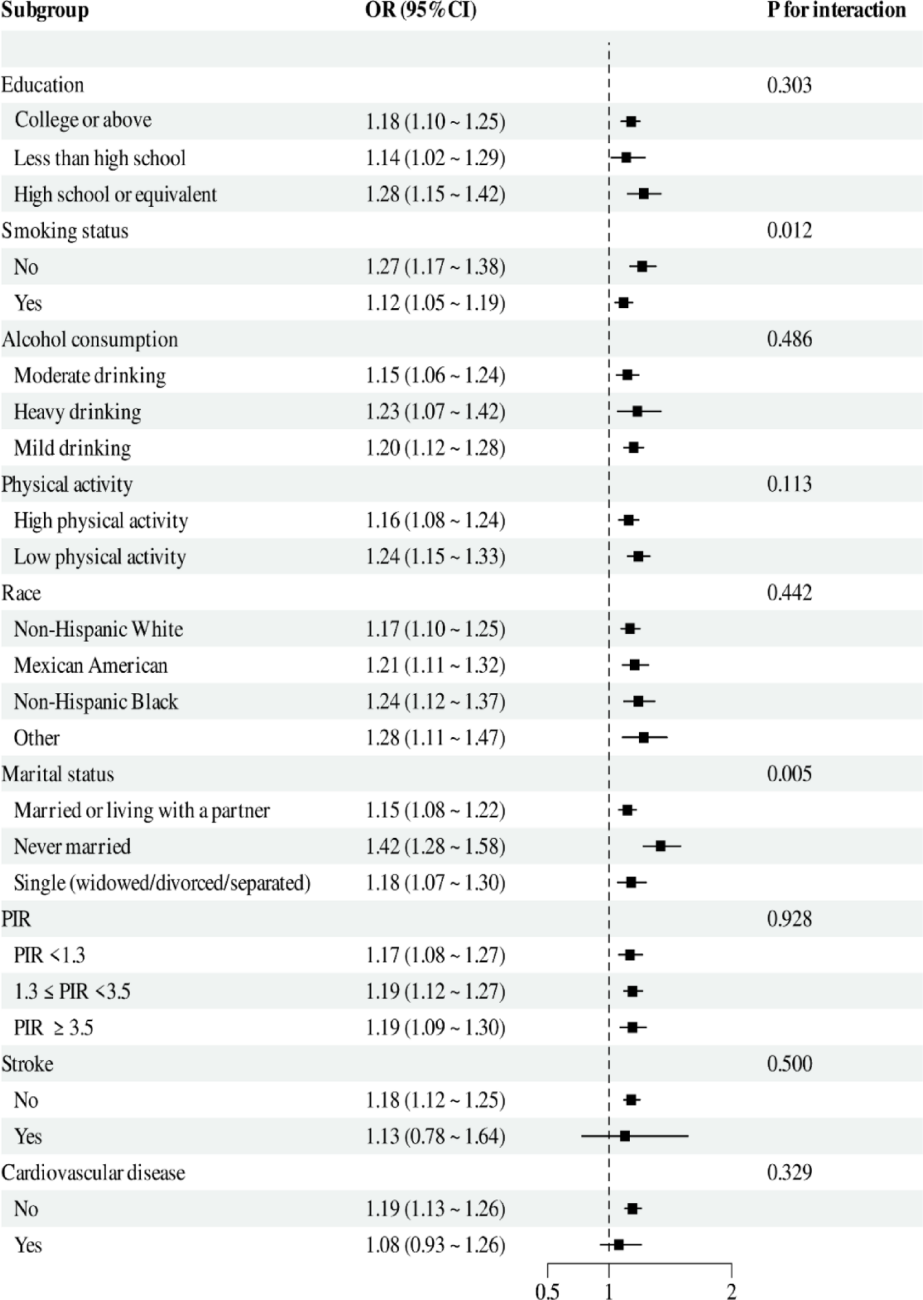
Subgroup analysis of FSI with OA.

In the subgroup analyses, significant interactions (*p* < 0.05) were observed between FSI and smoking status as well as marital status, whereas no evidence of interaction was found for education, alcohol consumption, physical activity, race, PIR, stroke, or cardiovascular disease (Fig. 3). Despite these interactions, higher FSI was consistently associated with OA prevalence across all subgroups.

### Mediation by phenotypic age

**Fig. 4:**
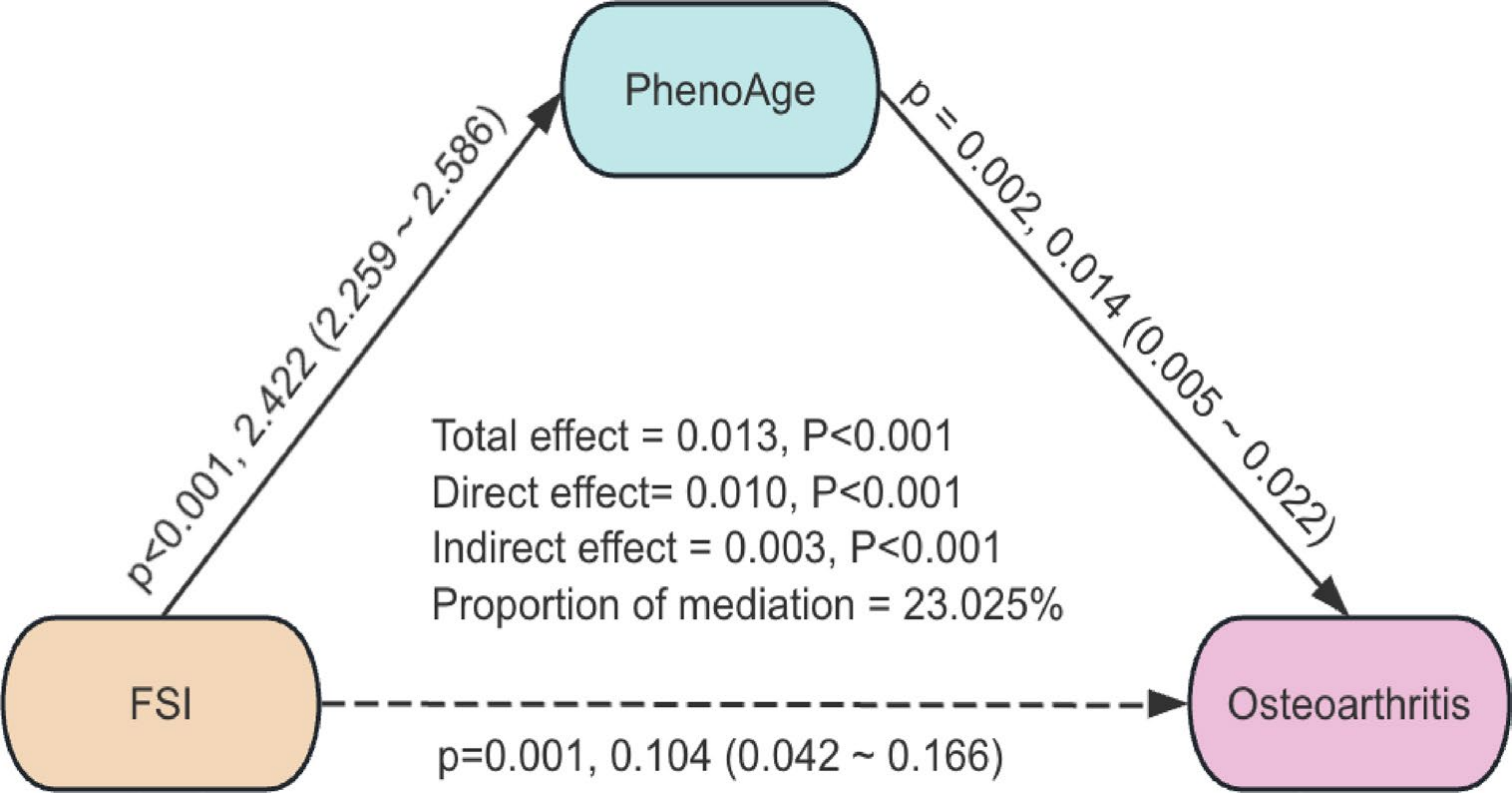
Path diagram of the exploratory mediation analysis of PhenoAge. Abbreviations: NHANES, National Health and Nutrition Examination Survey; CVD, Cardiovascular disease; AST, Aspartate Aminotransferase; GGT, Gamma-Glutamyl Transferase; ALT, Alanine Aminotransferase; TG, Triglycerides; FSI, Framingham steatosis index; BMI (kg/m^2^), Body mass index; PIR, Poverty income ratio; PhenoAge, Phenotypic Age; OA, osteoarthritis.

In the exploratory mediation analysis, PhenoAge was evaluated as a potential explanatory factor in the association between the FSI and OA. As shown in Fig. 4, the total association between FSI and OA was 0.013 (*p* < 0.001), of which 0.003 (*p* < 0.001) was attributed to the indirect association involving PhenoAge, corresponding to an indirect proportion of 23.03%. The direct association (0.010, *p* < 0.001) and the indirect association were both statistically significant, suggesting that PhenoAge may partially account for the observed association between FSI and OA. Given the cross-sectional design, these findings should be interpreted as exploratory and do not imply causal mediation.

## Discussion

In this study, higher FSI was significantly associated with OA prevalence, and this association persisted after multivariable adjustment. Exploratory mediation analysis suggested that PhenoAge may partially account for this association. Together, these findings indicate an association between metabolic steatosis–related profiles and OA at the population level.

Our findings are consistent with previous epidemiological studies reporting associations between fatty liver, metabolic dysfunction, and OA. Prior studies have observed positive correlations between NAFLD and OA or OA severity, supporting the presence of shared metabolic features across these conditions. For example, Lu et al. reported that NAFLD was positively correlated with OA in US adults and recommended OA patients be screened for NAFLD given the pandemic of these diseases^14^. Han et al. found that NAFLD was associated with knee osteoarthritis and disease severity^24^. Studies by Drapkina and Pacifico et al. suggest that metabolic abnormalities in the musculoskeletal system may be a common pathological pathway for NAFLD and OA^25,26^. Our study is the first, to our knowledge, to examine an NHANES-derived steatosis index (FSI) in relation to OA. Using FSI rather than imaging- or elastography-based measures enables the use of widely available clinical and laboratory data and may capture a composite metabolic-hepatic profile relevant to OA prevalence.

Although causal mechanisms cannot be inferred from this cross-sectional analysis, the observed associations may reflect shared metabolic and inflammatory features common to both fatty liver disease and OA. NAFLD is closely intertwined with insulin resistance, dyslipidemia, and chronic inflammation^27–30^. Elevated free fatty acids and pro-inflammatory adipokines from visceral fat and steatotic liver could contribute to cartilage catabolism and synovial inflammation^31,32^. For instance, obesity-driven inflammation and oxidative stress (e.g. IL-6, TNF-α) are known to degrade extracellular matrix and promote chondrocyte senescence^7^. Insulin resistance and hyperglycemia also have direct effects: high glucose levels induce advanced glycation end-products and inflammatory cytokines in joint tissues^33^. Notably, diabetes treatments (e.g. metformin) reduce BMI and have been observed to lower OA progression rates, suggesting an association between glucose metabolism and joint health^34,35^. Together, these observations suggest that NAFLD and OA may share overlapping metabolic and inflammatory processes.

PhenoAge captures cumulative physiological dysregulation across multiple biological systems and has been associated with a range of chronic conditions. In the present study, PhenoAge was statistically associated with both the FSI and OA prevalence. Exploratory mediation analysis suggested that PhenoAge may partially account for the observed association between FSI and OA. Chronic metabolic stress has been linked to cellular senescence, telomere attrition, and systemic frailty, processes that have been associated with musculoskeletal aging and OA in previous studies^36,37^. Prior work by He et al. reported that Phenotypic Age was associated with OA in the context of metabolic risk factors, supporting the notion that biological aging may represent a shared feature across multiple chronic conditions^21^. Together, these observations suggest that phenotypic aging may serve as a common correlate linking metabolic dysfunction and OA.

Strengths of this study include the use of a large, nationally representative dataset, which provides substantial statistical power and population-level relevance. By incorporating FSI and PhenoAge, this study explored associations between metabolic liver-related profiles, biological aging, and OA prevalence in a population-based setting. To our knowledge, this is the first study to examine the association between an NHANES-derived steatosis index and OA, and to explore the potential role of phenotypic aging in this relationship. These findings highlight the potential relevance of integrating metabolic and aging-related markers in OA research and may help inform future risk stratification and clinical investigations, while longitudinal studies are needed to clarify temporal relationships and causality.

However, our study has several limitations. First, the cross-sectional design precludes causal inference, and because all variables were measured at the same time point, the mediation analysis should be interpreted as exploratory rather than causal. Second, OA status was self-reported and may be subject to misclassification. Third, residual confounding cannot be fully excluded despite multivariable adjustment. Because the FSI integrates multiple metabolic components that are themselves associated with OA, the independent contribution of hepatic steatosis cannot be fully disentangled from its metabolic correlates, and potential circular reasoning should be considered when interpreting the findings. Fourth, detailed information on specific joint sites was not consistently available across NHANES cycles, precluding region-specific analyses. Finally, although NHANES is nationally representative of the U.S. population, the generalizability of these results to other populations may be limited.

## Conclusion

We found that the FSI was significantly associated with OA prevalence in U.S. adults. PhenoAge may partially account for this association, suggesting that metabolic and aging-related factors may be jointly related to OA. These findings highlight a potential link between metabolic status and biological aging in relation to OA and provide population-based evidence that may help inform future epidemiological research. Longitudinal and mechanistic studies are warranted to further clarify the temporal relationships and underlying biological processes involved.

## Data Availability

The survey data are publicly available on the Internet for data users and researchers throughout the world (www.cdc.gov/nchs/nhanes/).
